# Graphene Nanoribbon Gap Waveguides for Dispersionless and Low-Loss Propagation with Deep-Subwavelength Confinement

**DOI:** 10.3390/nano11051302

**Published:** 2021-05-14

**Authors:** Zhiyong Wu, Lei Zhang, Tingyin Ning, Hong Su, Irene Ling Li, Shuangchen Ruan, Yu-Jia Zeng, Huawei Liang

**Affiliations:** 1 Shenzhen Key Laboratory of Laser Engineering, College of Physics and Optoelectronic Engineering, Shenzhen University, Shenzhen 518060, China; 1800281004@email.szu.edu.cn (Z.W.); hsu@szu.edu.cn (H.S.); liling@szu.edu.cn (I.L.L.); scruan@szu.edu.cn (S.R.); yjzeng@szu.edu.cn (Y.-J.Z.); 2 Key Laboratory for Physical Electronics and Devices of the Ministry of Education and Shanxi Key Lab of Information Photonic Technique, School of Electronic Science and Engineering, Xi’an Jiaotong University, Xi’an 710049, China; eiezhanglei@mail.xjtu.edu.cn; 3 Shandong Provincial Engineering and Technical Center of Light Manipulations & Shandong Provincial Key Laboratory of Optics and Photonic Device, School of Physics and Electronics, Shandong Normal University, Jinan 250358, China; ningtingyin@sdnu.edu.cn

**Keywords:** graphene plasmons, dispersionless, deep-subwavelength gap, electro-optic switch, on-chip integration

## Abstract

Surface plasmon polaritons (SPPs) have been attracting considerable attention owing to their unique capabilities of manipulating light. However, the intractable dispersion and high loss are two major obstacles for attaining high-performance plasmonic devices. Here, a graphene nanoribbon gap waveguide (GNRGW) is proposed for guiding dispersionless gap SPPs (GSPPs) with deep-subwavelength confinement and low loss. An analytical model is developed to analyze the GSPPs, in which a reflection phase shift is employed to successfully deal with the influence caused by the boundaries of the graphene nanoribbon (GNR). It is demonstrated that a pulse with a 4 μm bandwidth and a 10 nm mode width can propagate in the linear passive system without waveform distortion, which is very robust against the shape change of the GNR. The decrease in the pulse amplitude is only 10% for a propagation distance of 1 μm. Furthermore, an array consisting of several GNRGWs is employed as a multichannel optical switch. When the separation is larger than 40 nm, each channel can be controlled independently by tuning the chemical potential of the corresponding GNR. The proposed GNRGW may raise great interest in studying dispersionless and low-loss nanophotonic devices, with potential applications in the distortionless transmission of nanoscale signals, electro-optic nanocircuits, and high-density on-chip communications.

## 1. Introduction

A perfect slow-light guiding system, concurrently holding the robust broadband dispersionless transmission, low loss, and deep-subwavelength mode confinement with a large refractive index, is highly desirable in modern nanophotonics [1]. Although both dispersionless and low-loss light-guiding can be simultaneously attained using all-dielectric structures [2,3], the mode sizes are comparatively large due to the diffraction limit. Surface plasmon polaritons (SPPs), propagating along the interface between plasmonic (e.g., metallic) and dielectric materials [4,5,6], are promising candidates for overcoming the diffraction limit and reducing the mode size to the deep-subwavelength scale [7,8,9,10,11]. The nanoscale confinement and low propagation loss of electromagnetic wave have been simultaneously realized using the hybrid plasmonic waveguide [12,13,14], metallic V-groove [15,16], metallic wedge [17,18], graphene monolayer [19,20], and so on [21,22,23,24,25,26]. However, the dispersion of SPPs in these waveguides is relatively high. So far, the dispersionless SPPs have mainly shown strong field confinement along the one-dimensional (1D) direction perpendicular to the interface between plasmonic and dielectric materials, while the mode fields are uniform in the other direction of cross sections [27,28]. Even regardless of the propagation loss, it is still very challenging to acquire broadband dispersionless SPPs with a two-dimensional (2D) deep-subwavelength confinement, owing to the strong frequency dispersion of common plasmonic materials [1]. Although some progress has been made using twisted bilayer plasmonic materials [29], multilayered axially uniform hybrid plasmonic-dielectric systems [1], and nonlinear graphene configurations [30,31], the robust dispersionless and deep-subwavelength guiding systems with low propagation loss are still very underdeveloped both in theory and experiment.

In this study, a graphene nanoribbon gap waveguide (GNRGW), which can be fabricated through a layer-by-layer stacking method [32], is proposed to realize the robust dispersionless light-guiding with a 2D deep-subwavelength confinement and low loss. An analytical model is established for the first time, to our knowledge, to study the gap SPPs (GSPPs) propagating in the GNRGW, in which the influence caused by the boundaries of the graphene nanoribbon (GNR) is dealt with by introducing a reflection phase shift. The analytical results agree well with the numerical simulations obtained using COMSOL Multiphysics. Significantly, the dispersion relation of the fundamental GSPP mode is linear within an ultra-wide spectral range (several microns in the frequency domain), which means that the broadband dispersionless propagation of light can be obtained. The corresponding mode width is only 10 nm, which is 3 orders of magnitude smaller than the free-space wavelength, and simultaneously the propagation length could be as long as 10 μm (175*λ*_GSPP_, *λ*_GSPP_ is the wavelength of the GSPPs). Furthermore, the dispersionless low-loss propagation with deep-subwavelength mode confinement has strong robustness against both the width change and the curving of GNRs. When a GNRGW array is employed as a multichannel optical switch, each channel can be controlled independently by tuning the chemical potential of the corresponding GNR via the gate voltage, even if the channel separation is as small as 40 nm. The proposed GNRGW not only possesses robust dispersionless and low-loss light-guiding properties with 2D deep-subwavelength field confinement, but also can be integrated with ultra-compact planar optoelectronic devices.

## 2. Theoretical Model and Method

**Figure 1:**
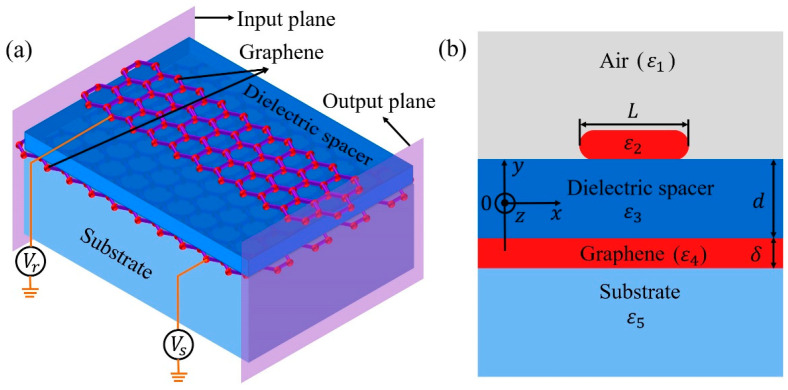
The schematic diagram of the GNRGW: (**a**) the 3D model; (**b**) the cross-sectional view in the *x*-*y* plane.

The GNRGW is schematically shown in Figure 1a. A GNR is separated from an underlying graphene sheet by a dielectric spacer with a thickness of *d* and a relative permittivity of *ε*_3_. The asymmetric structure is adopted to avoid the difficulty in alignment between two graphene layers, which facilitates the fabrication. Theoretically, the excellent tunability of graphene stems from the Pauli blocking of inter-band transitions [33,34]. The chemical potentials of the GNR and the graphene sheet can be modified by the applied voltages *V* [32,35]. The relationship between them can be written as $\mu_{c}=\mathrm{sgn}(n)\hbar v_{F}\sqrt{\pi \left|n\right|}$, where *v_F_* is the Fermi velocity and *n* = *C*_g_(*V* + *V*_0_)/*e* is the charge density. *C*_g_ and *V*_0_ are the gate capacitance and offset voltage, respectively [36]. The entire construction is embedded in the air with a relative permittivity of *ε*_1_ = 1, as shown in Figure 1b. The substrate with a relative permittivity of *ε*_5_ is semi-infinitely thick. To prevent unrealistically sharp edges, both terminations of the GNR are set to be semicircular with a curvature of *δ*/2, where *δ* = 0.34 nm is the thickness of graphene [37,38,39]. The relative permittivity of graphene, *ε*_g_, is calculated using *ε*_g_ = 1 + *iσ*_g_/(*ωε*_0_*δ*) [40,41], where *ε*_0_ and *ω* are the permittivity of vacuum and the angular frequency of the incident wave, respectively. The surface conductivity of graphene, *σ*_g_, can be evaluated using the Kubo formula [36,42,43], as shown in Figure S1 in Supplementary Materials A.

To analyze GSPPs guided by the GNRGW, the Helmholtz equation is solved using the method of separation of variables, and their dispersion equation is deduced as (see Supplementary Materials B)

(1) $$\frac{e^{2k_{2}\delta }(\xi_{1}+\xi_{2})\zeta_{A}+(\xi_{1}-\xi_{2})\zeta_{B}}{e^{2k_{2}\delta }(\xi_{1}+\xi_{2})\zeta_{C}+(\xi_{1}-\xi_{2})\zeta_{D}}=e^{2k_{4}\delta }\frac{\xi_{5}+\xi_{4}}{\xi_{5}-\xi_{4}},$$

where

(2) $$\zeta_{A}=e^{2k_{3}d}(\xi_{2}+\xi_{3})(\xi_{3}-\xi_{4})+(\xi_{2}-\xi_{3})(\xi_{3}+\xi_{4}),$$

(3) $$\zeta_{B}=e^{2k_{3}d}(\xi_{2}-\xi_{3})(\xi_{3}-\xi_{4})+(\xi_{2}+\xi_{3})(\xi_{3}+\xi_{4}),$$

(4) $$\zeta_{C}=e^{2k_{3}d}(\xi_{2}+\xi_{3})(\xi_{3}+\xi_{4})+(\xi_{2}-\xi_{3})(\xi_{3}-\xi_{4}),$$

(5) $$\zeta_{D}=e^{2k_{3}d}(\xi_{2}-\xi_{3})(\xi_{3}+\xi_{4})+(\xi_{2}+\xi_{3})(\xi_{3}-\xi_{4}),$$

*ξ_j_* = *k_j_*/*ε_j_* (*j* = 1, 2, 3, 4, or 5), *k_j_* = (*β*^2^ − *ε_j_k_0_*^2^ + *q_x_*^2^)^1/2^, and *k_0_* = (2π)/*λ*. *β* = *β*_1_ + *iβ*_2_ is the complex propagation constant along the *z*-axis; *λ* is the free-space wavelength; *ε_j_* is the relative permittivity at the corresponding region. *β*_2_ represents the loss factor of the propagated electromagnetic waves. The square root of the eigenvalue, *q_x_*, is influenced by the reflection on boundaries of the GNR, and the mode field distribution along the *x* direction, closely related to *q_x_*, is similar to that of a standing wave. To evaluate the influence of the boundaries on the GSPPs, a reflection phase shift, *φ*, is introduced for the first time, which satisfies the following boundary condition:

(6) $$q_{x}=\frac{m\pi -\varphi }{L},$$

where *m* is a natural number characterizing the mode order of the GSPPs, and *L* is the width of the GNR. The expression of *φ* is derived in detail in Supplementary Materials C (see Figure S2). When *φ* is determined, *q_x_* can be obtained. Then, *β* can be solved using Equation (1). The value of the reflection phase shift is negative for *m* = 0; thus, Equation (6) can still hold, which means that the fundamental mode can be guided. Significantly, the reflection phase shift can be employed to evaluate the boundary effect not only in GNRGW, but also in some other types of plasmonic waveguides with finite widths, such as the metal nanowire on a metal substrate [44,45] and the metal nanowire on graphene [46,47].

## 3. Results and Discussion

**Figure 2:**
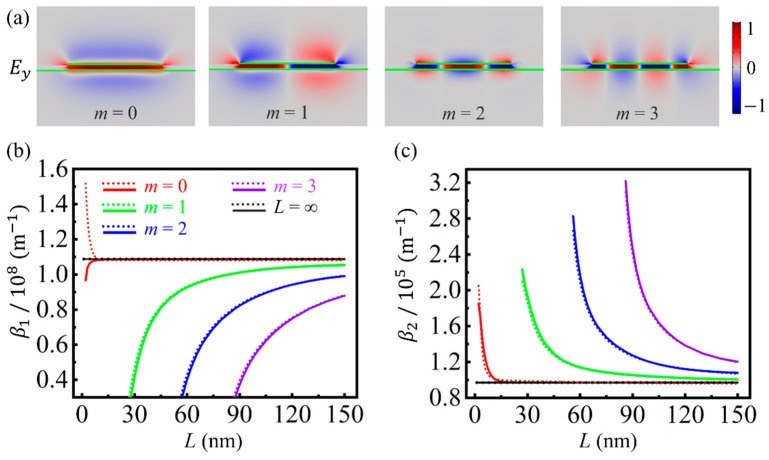
(**a**) Distributions of the *y*-components of electric fields, *E_y_*, for the four lowest-order GSPPs. The locations of graphene are indicated by the green lines. Dependences of the real (**b**) and imaginary (**c**) parts of *β* on *L* for the four modes. The solid and dashed lines correspond to the analytical model and simulation, respectively.

The field distributions of the four lowest-order GSPPs supported by the GNRGW with *L* = 120 nm are calculated using the commercially available finite element method package (COMSOL Multiphysics), as shown in Figure 2a. Other parameters are set to be: *ε*_3_ = 11.7 (silicon), *ε*_5_ = 2.25 (silica), *d* = 5.0 nm, both the chemical potentials of the GNR (*μ_cr_*) and graphene sheet (*μ_cs_*) are 1.0 eV, and the angular frequency of the incident wave *ω* = 1.78 × 10^14^ rad/s (or the equivalent wavelength *λ* = 10.6 μm), unless otherwise stated. According to the quantum-corrected model (QCM) unfolded in Supplementary Materials D (see Figures S3 and S4), the quantum tunneling effect is ignorable for a thickness of the dielectric spacer larger than 0.8 nm, which is also applicable to metallic nanocavities [48,49]. It can be seen that electromagnetic energy is mainly squeezed into the deep-subwavelength spacer between the two pieces of graphene. The *m*-th order mode has *m* field nodes with zero intensity along the *x* direction, and *E_y_* is symmetric and anti-symmetric for even-order and odd-order modes, respectively. Such GSPP modes could be excited by the near-field coupling method [50].

The dependences of real and imaginary parts of *β* on *L* are further calculated using both the analytical model and COMSOL Multiphysics, as shown in Figure 2b,c. *β*_1_ and *β*_2_ of GSPPs supported by infinitely extended double-layer graphene sheets (corresponding to *L* = ∞) are also provided for comparison, as shown by the black horizontal lines. The cutoff width of the GNR for each mode, *L_m_*, can be predicted by the analytical model (see Supplementary Materials C), where *β*_1_ and *β*_2_ tend to 0 and ∞, respectively. When *L* < 30 nm, all the high-order GSPPs (*m* ˃ 0) are cut off. For the fundamental mode (*m* = 0), *β* is independent on *L* in the range of *L* > 8 nm, which indicates its strong robustness against the width change. When *L* gets smaller from 8 nm, the fundamental GSPP mode gradually fades, and the edge mode gradually dominates (see Figure S5 in Supplementary Materials E). Thus, there is an obvious deviation between the analytical and simulated results in this range, as shown in Figure 2b, despite the fact that the proposed model is accurate for analyzing the GSPPs. In comparison with the fundamental mode, the complex propagation constants of the higher-order GSPPs are obviously affected by *L*.

**Figure 3:**
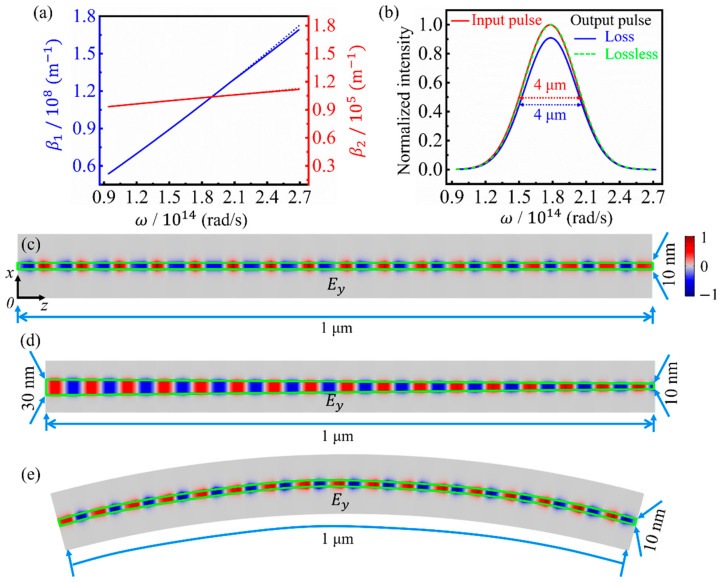
(**a**) Dependences of the real and imaginary parts of *β* on the angular frequency of incident wave for the fundamental GSPP mode. (**b**) The envelope profiles of pulses with a center wavelength of 10.6 μm and a FWHM of 4 μm in the frequency domain, probed on the input plane (red solid line) and the output plane (blue solid line), respectively. The green dashed line shows the pulse on the output plane without considering the propagation loss. Distributions of *E_y_* on the center planes of dielectric spacers (*y* = 0) in GNRGWs with the straight (**c**), wedge-shaped (**d**), and curved (**e**) GNRs, respectively, for *λ* = 10.6 μm. The interior areas of the green boxes represent GNRs. All electric field distributions share the same color legend.

The dispersion characteristics of guided modes are very important for both the fundamental principles and practical device-engineering [24], so the dependences of *β*_1_ and *β*_2_ on the angular frequency are calculated for the fundamental GSPP mode in a GNRGW with *L* = 10 nm. As shown in Figure 3a, the variation of *β*_2_ is very small; thus, the influence of the propagation loss on the dispersion is insignificant. The propagation length *L*_p_ = 1/*β*_2_ is approximately 10 μm (175*λ*_GSPP_ for *λ* = 10.6 μm), which is quite long for a guided mode with a 10 nm width. The small deviation between the simulated and analytical results in the high frequency range is caused by the evolution from GSPPs to edge plasmon modes (see Figure S5 in Supplementary Materials E). Notably, *β*_1_ increases linearly with *ω*, which means that the fundamental GSPP mode is broadband dispersionless. *β*_1_ is two orders of magnitude larger than the wave vector in free space, and the group velocity is *v_g_* = *dω*/*dβ*_1_ = *c*/180, where *c* is the speed of light in vacuum. The analytical relationship between *β*_1_ and *ω* is further discussed in Supplementary Materials F (see Figure S6).

To demonstrate the dispersionless and low-loss propagation of the deep-subwavelength GSPPs, the evolution of a pulse with a Gaussian envelope is simulated for a propagation distance of 1 μm. The normalized envelope profile of the input pulse with a center wavelength of 10.6 μm and a full width at half maximum (FWHM) of 4 μm in the frequency domain is described by the red solid line in Figure 3b. The peak value of the output pulse, as shown by the blue solid line in Figure 3b, is 10% smaller than that of the input pulse, owing to the inevitable propagation loss. However, if the amplitude of the output pulse is multiplied by 1.10 to ignore the propagation loss, its envelope profile, as shown by the green dashed line in Figure 3b, entirely coincides with that of the input pulse, which means a dispersionless propagation. The time-domain envelope profiles of the input and output pulses are also the same, as shown in Figure S7 in Supplementary Materials G. In the propagation process, the electromagnetic energy is well confined in the dielectric spacer covered by the GNR with a width of 10 nm, as shown in Figure 3c, so the mode width is approximately *λ*/1000. Therefore, all the propagation characteristics of the broadband dispersionless, low loss, and deep-subwavelength mode confinement can be attained simultaneously using the GNRGW.

To analyze the robustness of the dispersionless propagation with a deep-subwavelength mode width against the shape change of the GNR, the evolution of a broadband pulse propagating in a GNRGW with a wedge-shaped GNR is simulated. The distribution of *E_y_* indicates that the electromagnetic energy is always confined in the dielectric spacer covered by the GNR, and the mode width varies with *L*, as shown in Figure 3d. The electric field on the output plane is 1.39 times higher than that on the input plane; thus, the wedge-shaped structure can be used as an effective coupler between wide and narrow GNRGWs. To ignore the field enhancement caused by the focusing, the frequency- and time-domain amplitudes of the output pulse are multiplied by 0.72, as shown by the green dashed line in Figure S8a,b in Supplementary Materials H, and then the envelope profiles of the input and output pulses entirely coincide. Therefore, the dispersionless propagation in the GNRGW exhibits excellent robustness against the width change of the GNR. Furthermore, a GNRGW with a curved GNR is employed to guide the broadband pulse. The distribution of *E_y_* signifies that the electromagnetic energy can still be confined in the dielectric spacer covered by the curved GNR, as shown in Figure 3e. The amplitude of the output pulse is only 0.17% lower than that on the output plane of the straight GNRGW with the same length; thus, the bending loss is negligible. If the frequency- and time-domain amplitudes of the output pulse are multiplied by 1.10 to ignore the loss, as shown in Figure S9a,b in Supplementary Materials H, the envelope profiles completely coincide with those of the input pulse. Thus, the dispersionless propagation is also very robust against the curving of the GNR.

**Figure 4:**
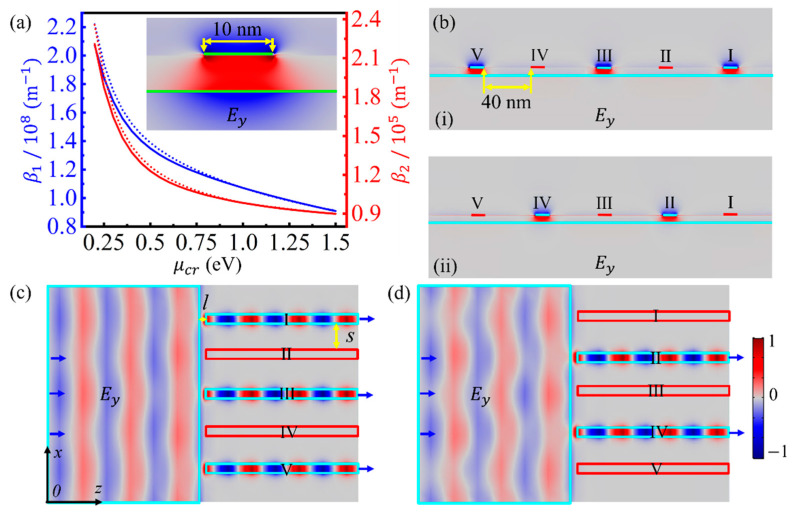
(**a**) Dependences of the real and imaginary parts of *β* on the chemical potential of the GNR. The inset in (**a**) shows the distribution of *E_y_*. (**b**) Distributions of *E_y_* on the output plane of a multichannel optical switch, parts (**i**) and (**ii**) corresponding to (**c**) and (**d**), respectively. (**c**,**d**) Distributions of *E_y_* on the center plane of the dielectric spacer for GNRs with different chemical potentials. The interior areas of the cyan and red boxes (or lines) represent graphene with chemical potentials of 1.0 and 0.3 eV, respectively. All electric field distributions share the same color legend.

The dependences of the real and imaginary parts of *β* on the chemical potential of the GNR are further investigated, as shown in Figure 4a. Both *β*_1_ and *β*_2_ decreases as the chemical potential increases, so the mode characteristics can be adjusted by modifying the gate voltage. *β*_1_ is much larger than the wave vector of light in vacuum, corresponding to a deep-subwavelength mode concentration, as shown in the inset of Figure 4a. Therefore, the GNRGWs can be employed as highly integrated photonic devices. Even if the separation between two adjacent GNRs is as small as 40 nm, the GSPPs can still propagate in each channel independently (see Figure S10 in Supplementary Materials I). Moreover, the dependences of *β* on *ε*_3_ and *ε*_5_ (see Figure S11 in Supplementary Materials J) indicate that the GSPPs can be guided by various GNRGWs with different spacers and substrates; thus, they are highly compatible with some other planar photonic devices.

The propagation of GSPPs in a GNRGW array with five GNRs is further simulated. The distributions of *E_y_* on the output plane of the array for GNRs with different chemical potentials are shown in Figure 4b, and the corresponding distributions on the center plane of the dielectric spacer are shown in Figure 4c,d, respectively. The left graphene sheet is used to couple light into the GNRGW array. The interval, *l*, is set to be 5 nm to separate the GNRs from the left graphene sheet, which ensures that the chemical potential of each GNR can be controlled independently by applying the gate voltage. According to Figure 2b, *β*_1_ of the fundamental GSPP mode is independent on the width of the GNR in the range of *L* > 8 nm; thus, the mode matching condition, *β*_1_, of GSPPs supported by the GNRGW on the right side and by the double-layer graphene sheets on the left side matching each other can be satisfied by applying the same gate voltage on the two parts. At this time, the light can be coupled into the corresponding GNRGW, resulting in an “on” state, as shown by I, III, and V channels in Figure 4c as well as II and IV channels in Figure 4d. When the mode condition is mismatched, the coupling efficiency decreases sharply, exhibiting an “off” state, as depicted by II and IV channels in Figure 4c as well as I, III, and V channels in Figure 4d. Both “on” and “off” states can be controlled independently by each GNRGW via adjusting the gate voltage, which allow the GNRGW array to be employed as high-density electro-optical switches with 20 channels per micron.

## 4. Conclusions

In conclusion, a GNRGW is proposed to realize robust dispersionless light-guiding with 2D deep-subwavelength field confinement and low propagation loss. An analytical model, which can successfully deal with the influence caused by the extremely narrow width of the GNR, is presented to analyze the GSPPs supported by the proposed GNRGW. It is demonstrated that when a pulse with a 10 nm mode size and a 4 μm bandwidth propagates over 1 μm in the GNRGW, no waveform distortion is observed, which is very robust against the shape change of the GNR. Furthermore, an electronically controlled multichannel optical switch is achieved by using a GNRGW array. Even if the separation between adjacent GNRGWs is as small as 40 nm, GSPPs in each channel can still be controlled independently by tuning the chemical potential of the corresponding GNR. The proposed GNRGW is expected to stimulate a new pathway to realize the highly integrated, broadband dispersionless photonic devices with deep-subwavelength mode confinement and low propagation loss.

## Data Availability

The data presented in this study are available on request from the corresponding author.

## Supplementary Materials

The following are available online at https://www.mdpi.com/article/10.3390/nano11051302/s1, Figure S1: Dependences of surface conductivity of graphene on the angular frequency of incident waves and the chemical potential of graphene, Figure S2: Schematic diagram of the reflection process, Figure S3: Dependences of *ε*_3_ on *d* obtained using the quantum-corrected model and the classical method, Figure S4: Dependences of *β* on *d* obtained using the quantum-corrected model and the classical method, Figure S5: The influence of *L* on the mode field distribution, Figure S6: Dependences of *β*_1_ and reflection phase shift on the angular frequency of incident waves, Figure S7: Time-domain envelope profiles of the input and output pulses in a GNRGW with a straight GNR, Figure S8: Frequency- and time-domain envelope profiles of the input and output pulses in a GNRGW with a wedge-shaped GNR, Figure S9: Frequency- and time-domain envelope profiles of the input and output pulses in a GNRGW with a curved GNR, Figure S10: Crosstalk analysis on two GNRGWs, Figure S11: Dependences of the complex propagation constant on the relative permittivities of the dielectric spacer and substrate.
