# Supplementary material for: Graphene Nanoribbon Gap Waveguides for Dispersionless and Low-Loss Propagation with Deep-Subwavelength Confinement
Source: Nanomaterials (Basel). 2021 May 14;11(5):1302. doi: 10.3390/nano11051302 (PMC8156105; doi:10.3390/nano11051302)
Supplement: Supplementary file 1 [file nanomaterials-11-01302-s001.zip › nanomaterials-1216145-supplementary.pdf]

SUPPLEMENTARY MATERIALS FOR:

Article

Graphene Nanoribbon Gap Waveguides for Dispersionless and Low-Loss Propagation with Deep-Subwavelength Confinement

Zhiyong Wu [1], Lei Zhang [2], Tingyin Ning [3], Hong Su [1], Irene Ling Li [1], Shuangchen Ruan [1], Yu-Jia Zeng [1] and Huawei Liang [1,*]

[1] Shenzhen Key Laboratory of Laser Engineering, College of Physics and Optoelectronic Engineering,
Shenzhen University, Shenzhen 518060, China; 1800281004@email.szu.edu.cn (Z.W.); hsu@szu.edu.cn (H.S.); liling@szu.edu.cn (I.L.L.); scruan@szu.edu.cn (S.R.); yjzeng@szu.edu.cn (Y.-J.Z.)

[2] Key Laboratory for Physical Electronics and Devices of the Ministry of Education and Shanxi Key Lab of Information Photonic Technique, School of Electronic Science and Engineering, Xi'an Jiaotong University, Xi'an 710049, China; eiezhanglei@mail.xjtu.edu.cn

[3] Shandong Provincial Engineering and Technical Center of Light Manipulations & Shandong Provincial Key Laboratory of Optics and Photonic Device, School of Physics and Electronics, Shandong Normal University, Jinan 250358, China; ningtingyin@sdnu.edu.cn

* Correspondence: hwliang@szu.edu.cn; Tel.: +86-755-8652-2081

A. Equations for calculating the surface conductivity of graphene

The optical response characteristics of graphene can be completely described by its surface conductivity, σ_g [1]. When both the external magnetic field and excitonic gap are zero, σ_g is isotropic [2] and its expression can be written as [2-4]

$$\sigma_g(\omega,\mu_c,\Gamma,T)=\sigma_{\text{intra}}(\omega,\mu_c,\Gamma,T)+\sigma_{\text{inter}}(\omega,\mu_c,\Gamma,T)\,, \tag{A1}$$

where the intra-band term of surface conductivity is

$$\sigma_{\text{intra}}(\omega,\mu_c,\Gamma,T)=\frac{ie^2k_BT}{\pi\hbar^2(\omega+i2\Gamma)}\left\{\frac{\mu_c}{k_BT}+2\ln[\exp(-\frac{\mu_c}{k_BT})+1]\right\}, \tag{A2}$$

the inter-band term of surface conductivity is

$$\sigma_{\text{inter}}(\omega,\mu_c,\Gamma,T)=\int_0^\infty\frac{ie^2(\omega+i2\Gamma)}{\pi\hbar^2}\frac{[\exp(\frac{-\Omega-\mu_c}{k_BT})+1]^{-1}-[\exp(\frac{\Omega-\mu_c}{k_BT})+1]^{-1}}{(\omega+i2\Gamma)^2-4(\Omega/\hbar)^2}d\Omega\,, \tag{A3}$$

ω is the angular frequency of the incident wave, $-e$ is the charge of an electron, $\hbar = h/(2\pi)$ is the reduced Planck's constant, k_B is Boltzmann's constant, and μ_c is the chemical potential of graphene. The temperature and charged particle scattering rate are set as $T = 300$ K and $\Gamma = 0.1$ meV, respectively.

σ_g can be flexibly picked by changing μ_c via the gate voltage, V. The dependence of μ_c on V can be expressed as $\mu_c = \text{sgn}(n)\hbar v_F(\pi|n|)^{1/2}$ [2], where v_F is the Fermi velocity and $n = C_g(V + V_0)/e$ is the charge density. C_g and V_0 are the gate capacitance and offset voltage, respectively.

Notably, σ_{inter} can be ignored when ω is too small to produce inter-band transitions in doped graphene. Specifically, the surface conductivity of graphene is dominated by the σ_{intra} for incident waves in the terahertz (THz) to mid-infrared spectral region, at room temperature, and under typical doping levels [1]. Furthermore, if the condition $\mu_c \gg k_BT$ is further fulfilled, the σ_g can be expressed by a Drude-like model as follows:

$$\sigma_g(\omega,\mu_c,\Gamma)=\frac{ie^2\mu_c}{\pi\hbar^2(\omega+i2\Gamma)}\,. \tag{A4}$$

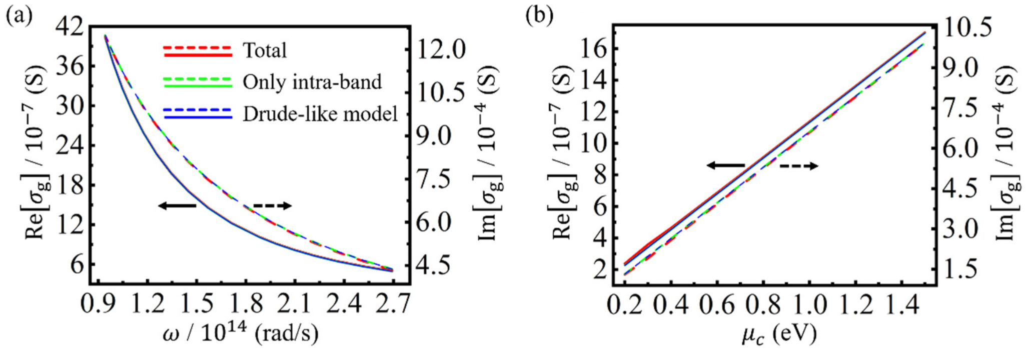

Figure S1. Dependences of surface conductivity of graphene on the angular frequency of incident waves (a) and the chemical potential of graphene (b), respectively. The red, green, and blue lines represent the surface conductivities calculated using the sum of intra-band and inter-band terms, only the intra-band term, and the Drude-like model, respectively. Moreover, the solid and dashed lines indicate the real and imaginary parts of the surface conductivity, respectively. $\mu_c = 1.0$ eV for (a) and $\omega = 1.78 \times 10^{14}$ rad/s for (b).

As shown in Figure S1, the red, green, and blue lines obtained using Eqs. (A1), (A2), and (A4),

respectively, are coincident; thus, the inter-band term is negligible, and the Kubo formula can be replaced by the Drude-like model for simplification. The Drude-like simplified model is very helpful for analytically studying both the quantum-corrected model (QCM) for the tunneling effect occurring in the extremely thin dielectric spacer and the dispersion relations of gap surface plasmon polaritons (GSPPs), which are presented in Sections **D** and **F**, respectively.

B. Derivations of the dispersion equation of GSPPs supported by the GNRGW

When two sheets of graphene are very close, the SPPs supported by graphene will couple with each other strongly and form the GSPPs. The GSPPs supported by the graphene nanoribbon gap waveguide (GNRGW) are the transverse magnetic (TM) modes, so the *x*-component of the magnetic field for GSPPs propagating along the *z*-direction can be written as

$$H_x(x,y,z,t) = f(x,y)e^{-i(\omega t-\beta z)}, \tag{B1}$$

where $\beta = \beta_1 + i\beta_2$ is the complex propagation constant. The corresponding Helmholtz equation can be written as

$$\nabla^2 H_x(x,y,z,t) + \varepsilon k_0^2 H_x(x,y,z,t) = 0, \tag{B2}$$

where ε is the relative permittivity of medium, $k_0 = (2\pi)/\lambda$ is the wavenumber, and λ is the free-space wavelength. Using the method of separation of variables, Eq. (B1) can be changed into

$$H_x(x,y,z,t) = X(x)Y(y)e^{-i(\omega t-\beta z)}. \tag{B3}$$

Substituting Eq. (B3) into Eq. (B2) yields

$$\frac{1}{Y(y)}\frac{\mathrm{d}^2Y(y)}{\mathrm{d}y^2} - (\beta^2 - \varepsilon k_0^2) = -\frac{1}{X(x)}\frac{\mathrm{d}^2X(x)}{\mathrm{d}x^2}. \tag{B4}$$

Since the left and right sides of the equal sign in Eq. (B4) are functions of the variables y and x, respectively, there is an underdetermined constant, q_x^2, that can make it true, namely

$$\frac{1}{Y(y)}\frac{\mathrm{d}^2Y(y)}{\mathrm{d}y^2} - (\beta^2 - \varepsilon k_0^2) = -\frac{1}{X(x)}\frac{\mathrm{d}^2X(x)}{\mathrm{d}x^2} = q_x^2. \tag{B5}$$

Eq. (B5) can be further decomposed into two equations:

$$\frac{\mathrm{d}^2X(x)}{\mathrm{d}x^2} + q_x^2 X(x) = 0, \tag{B6}$$

and

$$\frac{\mathrm{d}^2Y(y)}{\mathrm{d}y^2} - (\beta^2 - \varepsilon k_0^2 + q_x^2)Y(y) = 0. \tag{B7}$$

Electromagnetic fields are confined within the lateral range covered by the graphene nanoribbon (GNR), and the square root of the eigenvalue, q_x, can be solved by

$$q_x = \frac{m\pi - \varphi}{L}, \tag{B8}$$

where m is a natural number characterizing the number of wave-nodes with zero intensity along the *x*-direction, L is the width of the GNR, and φ is a phase shift caused by the reflection at the boundaries. The derivation related to φ is shown in Section **C**. Eq. (B8) ensures that when the GSPPs propagate over a period (with two reflections) along the *x*-direction, the phase change is $m2\pi$ for the m-th-order mode. In particular, for $m = 0$, the reflection phase shift with a negative value exactly compensates the phase q_xL in Eq. (B8), which ensures that the fundamental mode can be guided. The solution of Eq. (B7) can be written as

$$Y(y)=\begin{cases} A_1 e^{-k_1 y} & (d/2+\delta)<y \\ A_2 e^{k_2 y}+B_2 e^{-k_2 y} & d/2<y<(d/2+\delta) \\ A_3 e^{k_3 y}+B_3 e^{-k_3 y} & -d/2<y<d/2 \\ A_4 e^{k_4 y}+B_4 e^{-k_4 y} & (-d/2-\delta)<y<-d/2 \\ A_5 e^{k_5 y} & y<(-d/2-\delta) \end{cases}, \tag{B9}$$

where A_j (j = 1, 2, 3, 4, or 5) and B_l (l = 2, 3, or 4) are eight undetermined mode coefficients. $k_j = (\beta^2 - \varepsilon_j k_0^2 + q_x^2)^{1/2}$, in which ε_j is the relative permittivity at the corresponding region in Figure 1(b) (see the main manuscript). Based on Maxwell's equations, the electric field along z-axis of TM modes, E_z (x, y, z, t), can be solved using

$$E_z(x,y,z,t)=\frac{1}{i\omega\varepsilon_0\varepsilon}\frac{\partial H_x(x,y,z,t)}{\partial y}, \tag{B10}$$

where ε_0 is the permittivity of vacuum. By further employing the continuities of the tangential field components H_x (x, y, z, t) and E_z (x, y, z, t) at interfaces $y = d/2 + \delta$, $d/2$, $-d/2$, and $-d/2-\delta$, the following dispersion equation is obtained

$$\frac{e^{2k_2\delta}(\xi_1+\xi_2)\zeta_A+(\xi_1-\xi_2)\zeta_B}{e^{2k_2\delta}(\xi_1+\xi_2)\zeta_C+(\xi_1-\xi_2)\zeta_D}=e^{2k_4\delta}\frac{\xi_5+\xi_4}{\xi_5-\xi_4}, \tag{B11}$$

where

$$\zeta_A=e^{2k_3 d}(\xi_2+\xi_3)(\xi_3-\xi_4)+(\xi_2-\xi_3)(\xi_3+\xi_4), \tag{B12}$$

$$\zeta_B=e^{2k_3 d}(\xi_2-\xi_3)(\xi_3-\xi_4)+(\xi_2+\xi_3)(\xi_3+\xi_4), \tag{B13}$$

$$\zeta_C=e^{2k_3 d}(\xi_2+\xi_3)(\xi_3+\xi_4)+(\xi_2-\xi_3)(\xi_3-\xi_4), \tag{B14}$$

$$\zeta_D=e^{2k_3 d}(\xi_2-\xi_3)(\xi_3+\xi_4)+(\xi_2+\xi_3)(\xi_3-\xi_4), \tag{B15}$$

and $\xi_j = k_j/\varepsilon_j$.

C. Derivations of the reflection phase shift

When GSPPs propagate along the x-direction in a waveguide consisting of two infinitely extended graphene sheets (GSs), as shown in Figure S2(a), the main component of the magnetic field lies along the z-axis, which can be written as

$$H_z^I(x,y,t)=\begin{cases} C_1 e^{-h_1 y}\phi & (d/2+\delta)<y \\ (C_2 e^{h_2 y}+D_2 e^{-h_2 y})\phi & d/2<y<(d/2+\delta) \\ (C_3 e^{h_3 y}+D_3 e^{-h_3 y})\phi & -d/2<y<d/2 \\ (C_4 e^{h_4 y}+D_4 e^{-h_4 y})\phi & (-d/2-\delta)<y<-d/2 \\ C_5 e^{h_5 y}\phi & y<(-d/2-\delta) \end{cases}, \tag{C1}$$

where C_j (j = 1, 2, 3, 4, or 5) and D_l (l = 2, 3, or 4) are undetermined mode coefficients. $\phi = e^{-i(\omega t - px)}$ and $h_j = (p^2 - \varepsilon_j k_0^2)^{1/2}$, respectively, where p is the complex propagation constant along the x-direction. Based on Maxwell's equations, the y-component of the electric field can be obtained using

$$E_y^I(x,y,t)=\frac{p}{\omega\varepsilon_0\varepsilon}H_z^I(x,y,t)\cdot \tag{C2}$$

When the upper GS is replaced by a GNR of a finite width, the GSPPs arriving at the boundaries of the GNR will be reflected back into the lateral area covered by the GNR, as shown in Figure S2(b); thus, the guided GSPPs change into the localized gap plasmons (LGPs). The continuities of the tangential fields at the boundaries for calculating the complex reflection coefficient, *r*, can be written as [5]

$$(1+r)E_y^I = E_y^O, \tag{C3}$$

and

$$(1-r)H_z^I = H_z^O, \tag{C4}$$

where E_y^O and H_y^O are the *y*-component of the electric field and the *z*-component of the magnetic field outside the GNR, respectively. Expressing E_y^O in terms of Fourier-transformed quantities,

$$E_y^O = \int_{-\infty}^{\infty} g(k_y) e^{ik_y y} dk_y . \tag{C5}$$

By substituting Eq. (C3) into Eq. (C5), the expression of $g(k_y)$ can be written as

$$g(k_y) = \frac{1+r}{2\pi} I_1(k_y), \tag{C6}$$

where

$$I_1(k_y) = \int_{-\infty}^{\infty} E_y^I e^{-ik_y y} dy . \tag{C7}$$

Then, the magnetic field along *z*-axis can be written as

$$H_z^O = \frac{(1+r)\omega\varepsilon_0}{2\pi} \int_{-\infty}^{\infty} I_1 \frac{\varepsilon e^{ik_y y}}{\sqrt{\varepsilon k_0{}^2 - k_y{}^2}} dk_y . \tag{C8}$$

Taking the complex conjugate of Eq. (C4), multiplying it with Eq. (C3), and integrating the variable *y* yields

$$(1+r)(1-r^*)\int_{-\infty}^{\infty} E_y^I H_z^{I*} dy = \int_{-\infty}^{\infty} E_y^O H_z^{O*} dy = (1+r)\int_{-\infty}^{\infty} E_y^I H_z^{O*} dy, \tag{C9}$$

where

$$\int_{-\infty}^{\infty} E_y^I H_z^{O*} dy = \frac{(1+r^*)\omega\varepsilon_0}{2\pi} \int_{-\infty}^{\infty} I_1^* \int_{-\infty}^{\infty} \frac{E_y^I \varepsilon^* e^{-ik_y y}}{\sqrt{\varepsilon^* k_0{}^2 - k_y{}^2}} dy dk_y . \tag{C10}$$

Substituting Eq. (C10) into Eq. (C9), the complex reflection coefficient can be obtained as follows:

$$r = \left(\frac{1-G}{1+G}\right)^*, \tag{C11}$$

where

$$G = \frac{\omega\varepsilon_0}{2\pi} \frac{\int_{-\infty}^{\infty} I_1^* \int_{-\infty}^{\infty} \frac{E_y^I \varepsilon^* e^{-ik_y y}}{\sqrt{\varepsilon^* k_0{}^2 - k_y{}^2}} dy dk_y}{\int_{-\infty}^{\infty} E_y^I H_z^{I*} dy} . \tag{C12}$$

When *r* is determined, the reflection phase shift upon the boundaries, φ, can be solved using the following equation

$$\varphi = \arctan\left(\frac{r_2}{r_1}\right), \tag{C13}$$

where r_1 and r_2 are the real and imaginary parts of *r*, respectively. The cutoff width of the GNR for each mode can be further predicted by

$$L_m = \frac{m\pi - \varphi}{p}, \tag{C14}$$

which agrees well with the numerical simulation.

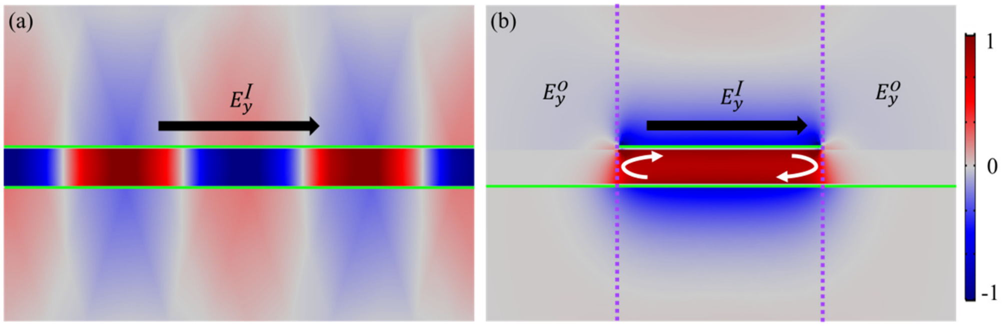

Figure S2. Distributions of the normalized electric field along the *y*-axis of GSPPs supported by the infinitely extended double-layer GSs (a) and the GNRGW (b), respectively. The locations of graphene are indicated by the green lines. This representation is also adopted in Figures S5 and S10(b).

D. QCM for the tunneling effect

The thickness of the dielectric spacer adopted is only a few nanometers; thus, the quantum tunneling effect of charged carriers across the spacer is further discussed using the QCM [6-10]. The quantum tunneling effect is manifested as inducing effective conductivity in the deep subwavelength nanogap and the dielectric spacer is regarded as a hypothetical metal with an effective electron density and resistance. Then, the dielectric function of the spacer is represented by a Drude-like model [9]:

$$\varepsilon_{3g}(d) = \varepsilon_3 + (\varepsilon_\infty - \varepsilon_3)e^{-\frac{d}{d_1}} - \frac{\omega_p{}^2}{\omega[\omega + i\gamma_g(d)]}, \tag{D1}$$

where $\varepsilon_{3\mathrm{g}}$ and ε_3 are the relative permittivities of the dielectric spacer with and without the quantum tunneling effect, respectively. ε_∞ and ω_p are the background permittivity and plasma frequency of graphene. The parameter $d_1 = 0.08$ nm. The damping rate, $\gamma_g(d)$, is a function of the spacer thickness, which can be expressed as

$$\gamma_g(d) = 2\Gamma e^{\frac{d}{d_2}}, \tag{D2}$$

where $d_2 = 0.04$ nm. Substituting Eq. (A4) into $\varepsilon_\mathrm{g} = 1 + i\sigma_\mathrm{g}/(\omega\varepsilon_0\delta)$,

$$\varepsilon_g = 1 - \frac{(e^2\mu_c)/(\varepsilon_0\delta\pi\hbar^2)}{\omega(\omega + i2\Gamma)}. \tag{D3}$$

The Drude model of the relative permittivity of graphene can be written as $\varepsilon_\mathrm{g} = \varepsilon_\infty - \omega_p{}^2/[\omega(\omega + i2\Gamma)]$; thus, $\varepsilon_\infty = 1$ and $\omega_p{}^2 = (e^2\mu_c)/(\varepsilon_0\delta\pi\hbar^2)$. Then, the dependences of the relative permittivity of the dielectric spacer on the thickness of the spacer can be calculated using Eq. (D1), as shown in Figure S3. When $d \geq 0.8$ nm, the blue and red lines coincide completely; thus, the quantum tunneling effect can be ignored.

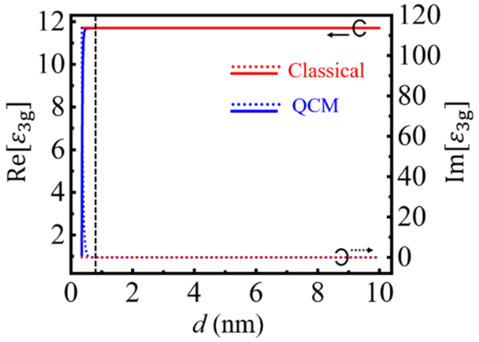

Figure S3. Dependences of the real (solid lines) and imaginary (dotted lines) parts of ε_{3g} on d. The blue and red lines are obtained by the QCM and the classical method (i.e., ignoring the quantum tunneling process), respectively.

The dependences of β on d are further calculated with and without considering the quantum tunneling effect, as shown in Figure S4. The two results agree well with each other for $d \geq 0.8$ nm. However, when $d < 0.8$ nm, both the real and imaginary parts of β acquired by the QCM are larger than the classical ones, which are consistent with the previously observed phenomena that the quantum tunneling effect is dominant in the sub-nanometer-scale dielectric gap [10, 11].

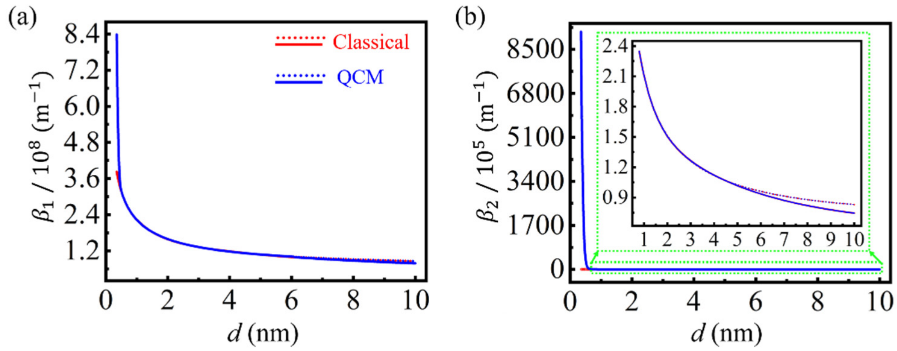

Figure S4. Dependences of the real (a) and imaginary (b) parts of β on d for the fundamental GSPP. The blue and red lines are obtained by the QCM and the classical method, respectively. The solid and dashed lines are obtained by the analytical model and COMSOL Multiphysics, respectively. This notation is also used in Figures S6(a) and S11. The enlarged image in (b) is a close-up view when the quantum tunneling effect can be ignored.

E. Evolutions from GSPPs to edge plasmon modes

The distributions of the normalized electric fields, |***E***|, for GNRs with different widths are calculated for a given spacer thickness $d = 5.0$ nm, as shown in Figure S5. When L decreases from 10 to 2 nm, the GSPPs sustained in the dielectric spacer covered by the GNR gradually fade and the edge plasmon mode with more energy concentrated at the ends of the GNR becomes dominant. Thus, the deviation between the analytical and simulated results increases gradually. The geometry of the GNRGW is unchanged along the propagation direction; thus, the mesh on the cross section is constructed and then swept along the propagation direction in the simulation. The graphene layer on the cross section is first divided along its boundary and then the "mapping" method is used to divide the mesh of the surface.

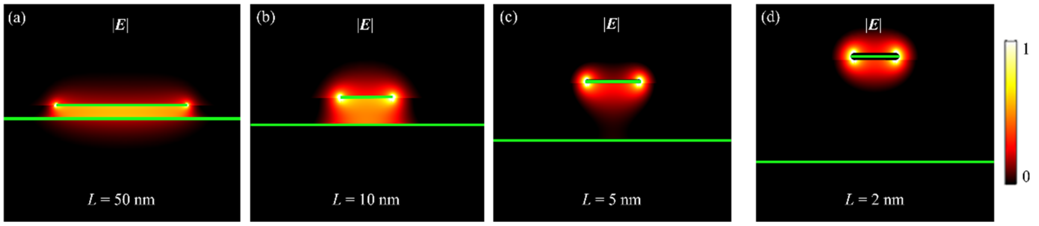

Figure S5. Distributions of the normalized electric fields for L = 50 nm (a), L = 10 nm (b), L = 5 nm (c), and L = 2 nm (d), respectively.

F. Simplifications of the dispersion equation of the GSPPs

Graphene is modeled as an infinitely thin surface characterized by its surface conductivity, σ_g, and its thickness, δ, is ignored to simplify the dispersion equation (B11), which can be written as [1]

$$\frac{\left(\frac{\varepsilon_5}{k_5}+i\frac{\sigma_g}{\omega\varepsilon_0}-\frac{\varepsilon_3}{k_3}\right)\left(\frac{\varepsilon_1}{k_1}+i\frac{\sigma_g}{\omega\varepsilon_0}-\frac{\varepsilon_3}{k_3}\right)}{\left(\frac{\varepsilon_5}{k_5}+i\frac{\sigma_g}{\omega\varepsilon_0}+\frac{\varepsilon_3}{k_3}\right)\left(\frac{\varepsilon_1}{k_1}+i\frac{\sigma_g}{\omega\varepsilon_0}+\frac{\varepsilon_3}{k_3}\right)}=e^{2k_3 d}, \tag{F1}$$

where it has taken the same natures for the GNR and GS. To further simplify Eq. (F1), $\varepsilon_1=\varepsilon_3=\varepsilon_5$ is adopted, then

$$2\frac{\varepsilon_3}{k_3}+i\frac{\sigma_g}{\omega\varepsilon_0}\left(1-e^{-k_3 d}\right)=0. \tag{F2}$$

By taking the approximation of $1-e^{-k_3 d}\approx k_3 d$, Eq. (F2) can be reduced to

$$\beta_1=\sqrt{\left(\frac{2\pi\hbar^2}{e^2\mu_c d}+\mu_0\right)\varepsilon_0\varepsilon_3\omega^2-q_x^2}, \tag{F3}$$

where the charged particle scattering rate of graphene is ignored and the discussion is restricted to the real part of β. $\left(\frac{2\pi\hbar^2}{e^2\mu_c d}+\mu_0\right)\varepsilon_0\varepsilon_3\omega^2 \gg q_x^2$ holds for the fundamental GSPPs (m = 0); thus, Eq. (F3) can be written as

$$\beta_1=\omega\sqrt{\left(\frac{2\pi\hbar^2}{e^2\mu_c d}+\mu_0\right)\varepsilon_0\varepsilon_3}. \tag{F4}$$

Eq. (F4) clearly shows that β_1 increases linearly with ω; thus, the corresponding group velocity $v_g = d\omega/d\beta_1$ is a constant. The analytical results (red solid line) calculated using Eq. (F4) are consistent with the simulated results (red dashed line) obtained using COMSOL Multiphysics, as shown in Figure S6(a). Since the electromagnetic energy is mainly concentrated in the dielectric spacer with a fixed refractive index between the two sheets of graphene, the GSPPs exhibit dispersionless propagation characteristics. The reflection phase shift, φ, is much smaller than π, as shown in Figure S6(b); thus, q_x for higher-order GSPPs is much larger than that for the fundamental mode. The dispersion behavior of the first-order mode (green solid line) is calculated using Eq. (F3), which also agrees well with the simulated results (green dashed line).

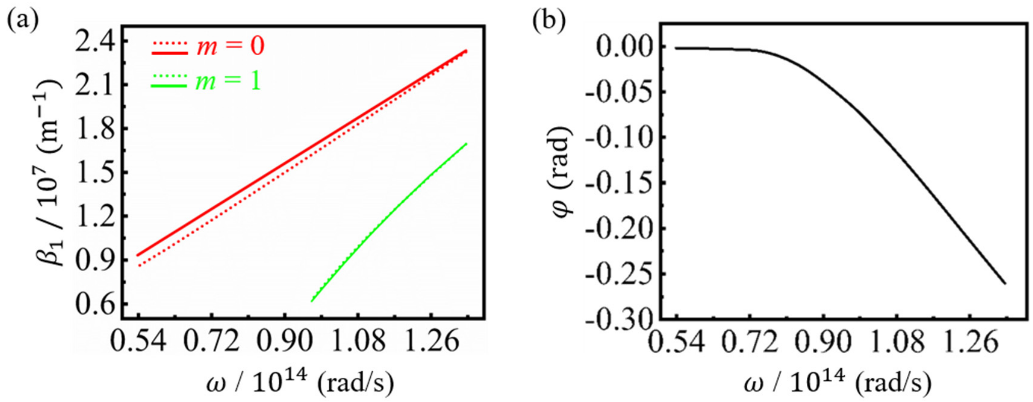

Figure S6. Dependences of β_1 (a) and reflection phase shift (b) on the angular frequency of incident waves. The width of GNR is L = 200 nm and the relative permittivities of the dielectric materials are adopted as $\varepsilon_1 = \varepsilon_3 = \varepsilon_5 = 1$.

G. Time-domain envelope profiles of the pulses in a GNRGW

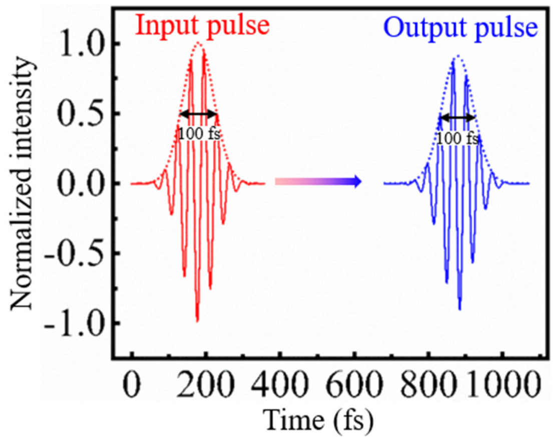

Figure S7. Time-domain envelope profiles of the input and output pulses in a GNRGW with a straight GNR. The amplitude of the output pulse is 10% smaller than that of the input pulse, while their full widths at half maximum (FWHMs) are the same, which agree well with the results in the frequency domain.

H. Robustness of the GNRGW against the GNR shape

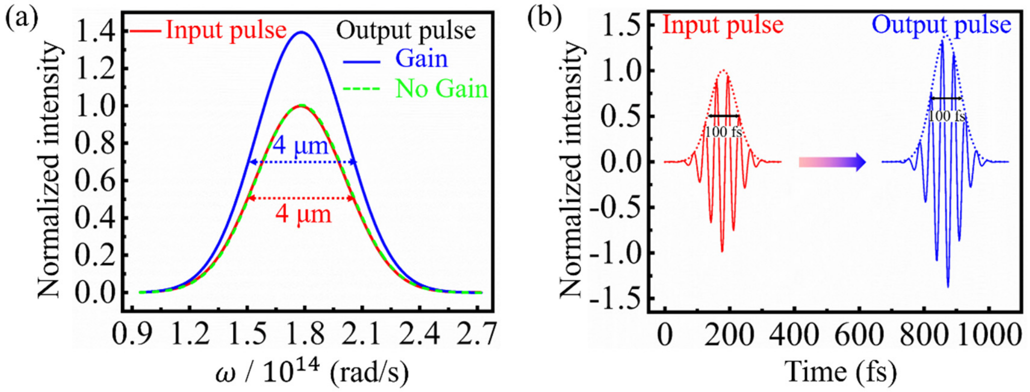

Figure S8. Frequency- (a) and time-domain (b) envelope profiles of the input and output pulses in a GNRGW with a wedge-shaped GNR.

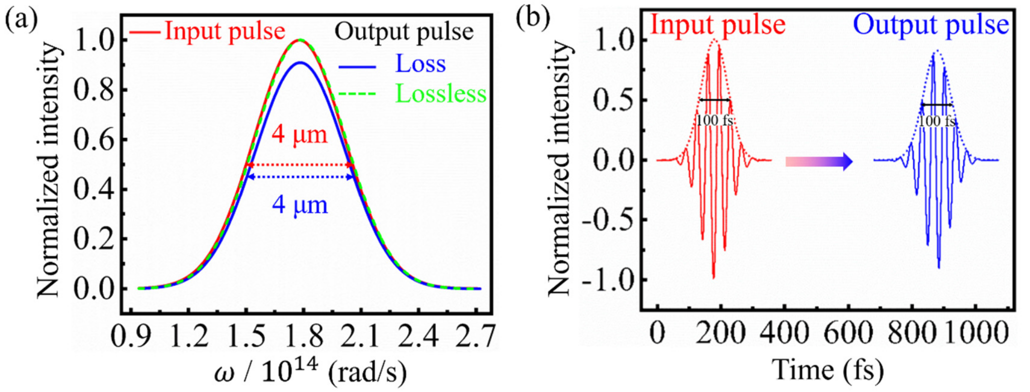

Figure S9. Frequency- (a) and time-domain (b) envelope profiles of the input and output pulses in a GNRGW with a curved GNR.

I. Crosstalk analyses for the GNRGW

The crosstalk of the GNRGW is further analyzed by replacing the GNR in Figure 1 in the main manuscript with two identical GNRs, whose cross section is shown in Figure S10(a). GSPPs parking in the dielectric spacer under the two GNRs can couple with each other to form the symmetric and anti-symmetric modes, as shown in Figure S10(b). To quantify the coupling strength between the two channels, the dependences of β of the two modes on the separation, s, are shown in Figure S10(c). As s decreases, the coupling strength increases, resulting in an increased difference between β of the two modes.

The coupling length, L_C, defined as $L_C = \pi/|\beta_{1,s} - \beta_{1,as}|$ [12, 13], is applied to measure the crosstalk, which is the required length for transferring all the power from one channel to the other. $\beta_{1,s}$ and $\beta_{1,as}$ represent the real parts of β of the symmetric and anti-symmetric modes, respectively. The dependence of the normalized coupling length, L_C/L_p, on s is shown in Figure S10(d), where L_p denotes the propagation length of the GSPPs without the coupling effect. Generally, the coupling between two

adjacent channels can be ignored when $L_C/L_p > 1$ [13]. L_C/L_p reaches 1 at $s \approx 35$ nm; thus, the GNRGW array with a channel separation of 40 nm in the main manuscript shows extremely weak crosstalk between adjacent components.

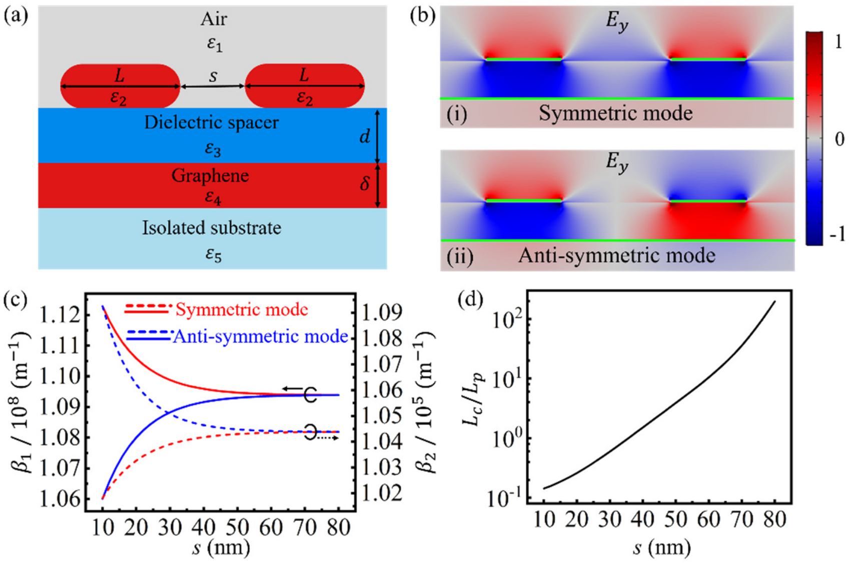

Figure S10. (a) The cross section of the proposed coupling configuration. (b) The distributions of normalized electric fields along y-axis for the symmetric (i) and anti-symmetric (ii) modes. Dependences of the complex propagation constant (c) and normalized coupling length (d) on the separation between two GNRs.

J. Modes properties with respect to medium's permittivity

The dependences of the complex propagation constant on relative permittivities of the dielectric spacer and substrate are shown in Figures S11(a) and (b), respectively. Both β_1 and β_2 increase as the relative permittivities increase, but ε_3 has a much greater impact on β than ε_5, owing to most of the electromagnetic energy being concentrated in the dielectric spacer. The GSPPs can be guided by various GNRGWs with different spacers and substrates, which indicates their good compatibility with other planar photonic devices.

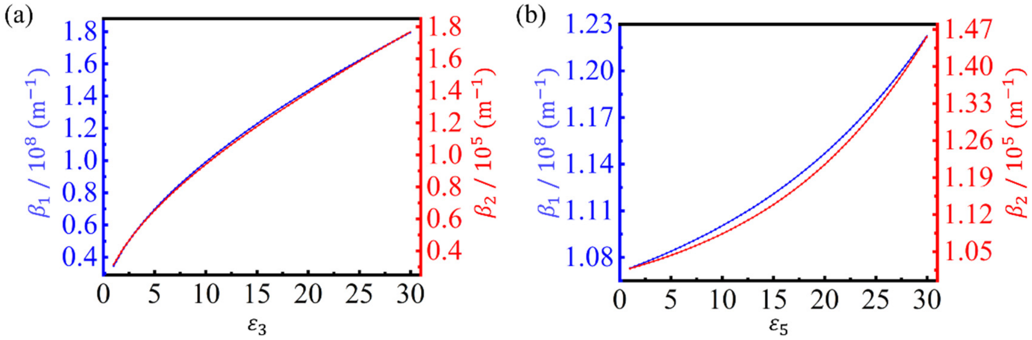

Figure S11. The dependences of the complex propagation constant on the relative permittivities of the dielectric spacer (a) and substrate (b), respectively.

References

1. P. A. D. Gonçalves and N. M. R. Peres, *An introduction to graphene plasmonics* (World Scientific, 2016).
2. H. W. Liang, L. Zhang, S. Zhang, T. Cao, A. Alu, S. C. Ruan, and C. W. Qiu, "Gate-programmable electro-optical addressing array of graphene-coated nanowires with sub-10 nm resolution," ACS Photonics **3**, 1847-1853 (2016).
3. V. P. Gusynin, S. G. Sharapov, and J. P. Carbotte, "Magneto-optical conductivity in graphene," J. Phys.-Condes. Matter **19**, 026222 (2007).
4. G. W. Hanson, "Dyadic Green's functions and guided surface waves for a surface conductivity model of graphene," J. Appl. Phys. **103**, 064302 (2008).
5. A. Chandran, E. S. Barnard, J. S. White, and M. L. Brongersma, "Metal-dielectric-metal surface plasmon-polariton resonators," Phys. Rev. B **85**, 085416 (2012).
6. R. Esteban, A. G. Borisov, P. Nordlander, and J. Aizpurua, "Bridging quantum and classical plasmonics with a quantum-corrected model," Nat. Commun. **3**, 825 (2012).
7. L. Wu, H. G. Duan, P. Bai, M. Bosman, J. K. W. Yang, and E. P. Li, "Fowler-nordheim tunneling induced charge transfer plasmons between nearly touching nanoparticles," ACS Nano **7**, 707-716 (2013).
8. S. F. Tan, L. Wu, J. K. W. Yang, P. Bai, M. Bosman, and C. A. Nijhuis, "Quantum plasmon resonances controlled by molecular tunnel junctions," Science **343**, 1496-1499 (2014).
9. R. Esteban, A. Zugarramurdi, P. Zhang, P. Nordlander, F. J. Garcia-Vidal, A. G. Borisov, and J. Aizpurua, "A classical treatment of optical tunneling in plasmonic gaps: extending the quantum corrected model to practical situations," Faraday Discuss. **178**, 151-183 (2015).
10. D. J. Yang, S. P. Zhang, S. J. Im, Q. Q. Wang, H. X. Xu, and S. W. Gao, "Analytical analysis of spectral sensitivity of plasmon resonances in a nanocavity," Nanoscale **11**, 10977-10983 (2019).
11. R. Esteban, G. Aguirregabiria, A. G. Borisov, Y. M. M. Wang, P. Nordlander, G. W. Bryant, and J. Aizpurua, "The morphology of narrow gaps modifies the plasmonic response," ACS Photonics **2**, 295-305 (2015).
12. G. Veronis and S. Fan, "Crosstalk between three-dimensional plasmonic slot waveguides," Opt. Express **16**, 2129-2140 (2008).
13. K. Zheng, Y. F. Yuan, L. T. Zhao, Y. Chen, F. Zhang, J. Song, and J. L. Qu, "Ultra-compact, low-loss terahertz waveguide based on graphene plasmonic technology," 2D Mater. **7**, 015016 (2020).
